# The core values and principles of public health: a contemporary treatise

**DOI:** 10.3389/fpubh.2026.1919058

**Published:** 2026-09-04

**Authors:** Georgia Efthymiou, Rafael Omirou, Eleftheria C. Economidou, Elpidoforos S. Soteriades

**Affiliations:** 1 Healthcare Management Program, School of Economics and Management, Open University of Cyprus, Nicosia, Cyprus; 2 Department of Pediatrics, Larnaca General Hospital, Larnaca, Cyprus

**Keywords:** inequalities, policies, principles, programs, public health, values

## Abstract

Public health constitutes an inclusive term with contested boundaries difficult to define in universally accepted terms. There is an unceasing debate on the narrowness or broadness of public health definition, in relation to corresponding policies, actions and/or inactions implemented in the field. In this treatise, based on a narrative review methodology, we aimed at developing a new framework of core public health values and principles using specific criteria and building on prior published work. Our proposed framework includes an integrated set of five fundamental public health values (respect, beneficence, non-maleficence, justice and solidarity), and five principles (accessibility, affordability, acceptability, effectiveness and participation), which highlight the way these moral compasses lead to the development of associated programs and policies. A paradigm delineated by the Patient protection and Affordable Care Act (ACA), aligns such values and principles towards improving society’s health outcomes, social engagement and reduction of health inequalities, along with healthcare systems’ resilience. Focus is also being given to epidemiology as a facilitator of public health interventions to achieve universal health coverage and decision making that incorporates public health values and principles in everyday life. The integration of values and principles enables the development of a comprehensive framework, which links policies and programs with individual and collective needs, particularly regarding poor and socially vulnerable populations. By upholding these values and principles as our main public health compasses, we could mitigate health disparities, address current public health challenges and ensure resilient, equitable and sustainable healthcare systems for all.

## Introduction

Public health is an inclusive term with contested boundaries, difficult to define in widely accepted terms. It is also quite challenging to characterize public health within a set of specific conceptual borders since it ranges from the narrowest to the broadest definitions (1). The most famous definition of Public health has been offered by Dr. Winslow in 1920 stating that “Public health is the science and art of preventing disease, prolonging life and promoting health and efficiency through organized community efforts for the sanitation of the environment, the control of communicable infections, the education of the individual in personal hygiene, the organization of medical and nursing services for the early diagnosis and preventive treatment of disease, and for the development of the social machinery to insure everyone a standard of living adequate for the maintenance of health, so organizing these benefits as to enable every citizen to realize his birthright of health and longevity” (2).

Public health is concerned with the well-being of both individuals and populations. Since the late 1990s, numerous public health ethical frameworks have emerged, ranging from extensions of principle-based models to human rights and social justice perspectives, to those based on political philosophy. While no single framework has been universally adopted within the field of public health, public health professionals argue considerably about the broadness of public health definition mainly because defining the limits of public health constitutes the most fundamental criterion that guides the corresponding policies, programs and actions or inactions implemented by public health professionals, educators and policy makers (3). The complexity of current public health problems in parallel to the re-emergence of long forgotten infectious diseases along with the increasing epidemic of non-communicable diseases and global social unrest with acute population migrations due to war and social conflict, constitute a difficult complex mixture that may require the adoption of the broadest definition of public health to employ effective interventions to mitigate the challenges of our current era (4, 5). Such a broad and inclusive approach requires a solid definition and theoretical foundation and plays a critical role in shaping the structure, guiding the content, and assessing the implementation and outcomes of public health policies and programs. In addition, it could also be in direct concordance with the adoption of the definition of health by WHO as the state of complete physical, mental and social well-being and not merely the absence of disease or infirmity and also comply with contemporary discussions on the one health concept (6). This is an invaluable asset that enables individuals to lead fulfilling lives, and enhances productivity, while it promotes national growth and development.

**Figure 1:**
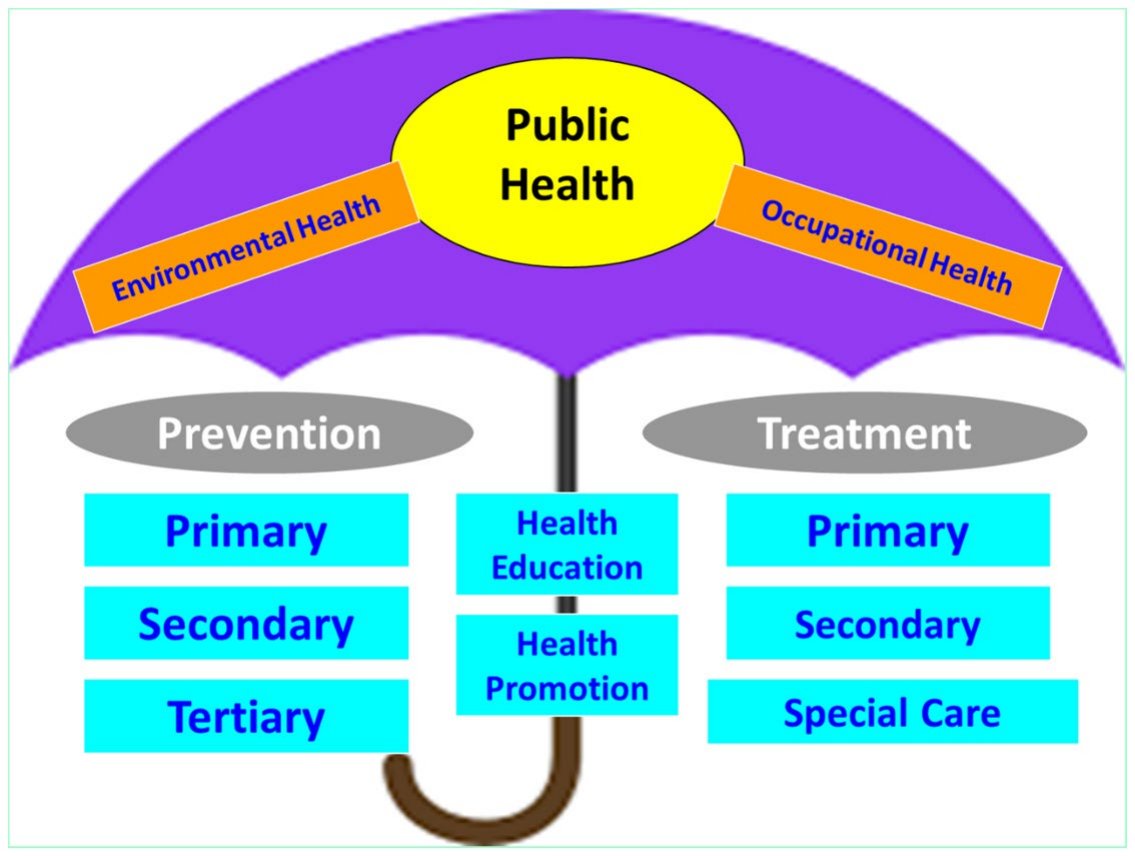
The public health umbrella paradigm.

Public health incorporates theory and practice, aiming to achieve four major societal goals, which public health professionals and policy makers aim to achieve through evidence-based organized efforts of the society, namely: protect and promote health and well-being, prevent disease and disability, prolong life expectancy, and maintain and improve quality of life for the population as a whole. Public health is an umbrella term under which we strive to include two main pillars, prevention and treatment (healthcare services) (7). The first pillar of Public Health is instrumentally supported by a number of national health agencies, which provide the necessary network of programs and services to facilitate not only primary, secondary and tertiary prevention but also health education and health promotion as well as provide the appropriate interchange and interaction with the second pillar of quality healthcare services. The second pillar of public health refers to the institutions that deal with disease management for individuals and populations alike. It includes emergency and primary healthcare services, secondary treatment (hospital care) and additional specialized (tertiary) care centers (Figure 1).

Preventive medicine is a specialized field that gives high priority to overall health by promoting individual services but also focuses on population-based programs. Philosophy pivots around health as a critical asset that not only improves quality of life but also enhances productivity for individuals and societies (8). Often, public health is misunderstood as synonymous with preventive medicine, due to sharing the overarching goal of improving and safeguarding community health through the formulation of policies and programs that aim to prevent health problems by engaging both health professionals and other non-physician experts. However, public health is highly valued in this process, since it involves designing, adapting, implementing, and assessing population-based interventions in different settings (9).

Values serve as the overarching ethical foundation of public health, representing the core ethical ideals and societal commitments. A parallel symbolism is that values function much like as the “theoretical framework” which paves the conceptual pathway guiding our social engagement, while principles, help translate the abstract ethical commitments embedded in values into more specific, action-oriented practices that help navigate decision-making into everyday life (10, 11) (Table 1). These principles then inform the development of concrete policies and programs, which represent the practical application of public health ethics in social life. Principles represent action guides that offer focused guidance which are broader than those provided by rules, regulations and guidelines. According to Gert and colleagues, principles encapsulate an entire moral theory (values), providing a concise reference to assist moral agents in making ethical decisions in applying action guides that depend on the moral theory to which one subscribes (12–14). Principles represent how we act (e.g., operational guidance). In contrast, values represent why we act; they reflect the underlying motivations, beliefs, and ethical commitments that drive public health choices.

**Table 1:** Key features of proposed core values and principles of public health.

Description of values and principles	Key features	References
Core values		
Respect	Recognition of human dignity and autonomy with informed consent. Protection of individual rights, including the right of knowing, privacy and informed decision-making. Special support for individuals with diminished autonomy (e.g., children, older adults, disabled)	(19, 36)
Beneficence	Moral obligation to promote the well-being of individuals and communities. Development of policies that maximize benefits and minimize harm. Advocacy for vulnerable populations and promotion of social determinants of health.	(13, 19, 20, 51)
Non-Maleficence	First do no harm. Responsibility to avoid harm through actions or omissions. Balancing potential risks and benefits of public health interventions. Ethical decision making in surveillance, screening, regulation and health communication.	(21, 51)
Justice	Fair distribution of health resources and opportunities. Equal access to preventive and therapeutic services regardless of socioeconomic or personal characteristics. Reduction of health disparities and structural inequalities.	(19, 22, 51)
Solidarity	Mutual support and shared responsibility for health. Collective actions that promote social cohesion, inclusive public health policies and equitable healthcare access. Health is non-competitive, non-rivalrous and a non-excludable access good. In general, one person’s health does not diminish another’s. Emphasis on community bonds, networks and cooperation.	(13, 22–24)
Core principles		
Accessibility	Ensuring timely and equitable access to essential health services. Elimination of geographic, financial and systemic barriers to care and programs. Promoting preventive care and reaching underserved populations.	(25, 27)
Affordability	Access to healthcare without financial hardship. Investment in preventive services to reduce long-term, out of pocket and hidden costs. Policies that reduce personal expenses and mitigate economic disparities.	(29)
Acceptability	Alignment of healthcare services with age, gender, cultural, social, religious and ethical values of communities. Community engagement and culturally sensitive interventions that respect community beliefs and practices.	(UN Doc. E/C. CESCR General Comment No. 14/2000) (30)
Effectiveness	Implementation of evidence-based, fail-proof and outcome-driven, cost-effective public health interventions. Continuous monitoring and adaptation to ensure measurable improvements in population health.	(31, 32)
Participation	Active community involvement in the design, implementation and evaluation of public health programs. Bottom-up approach—empowerment of local populations and collaborative decision-making for sustainable health outcomes.	(33)

Public health ethics represent a systematic approach to clarify, prioritize, and justify possible courses of public health action. These actions are based on ethical values and principles, while they are shaped by scientific evidence. It is widely accepted that ethical tools used to support related decisions in public health are not identical with those applied in clinical medicine (bioethics and medical ethics), which are more individual and patient-oriented (15–17).

These precise ethical values and principles have been a topic of vigorous discussion since public health ethics splintered off from clinical ethics in the mid-1980s. Thus far, we have not yet arrived at a consensus theory, framework, or approach to solidify modern public health ethics (11, 17, 18). The development of an agreed-upon set of core values and principles in the field of public health remains an ongoing debate even today. A value- and principle-based approach to public health facilitates advocating for people-centered health systems, promoting health throughout the life-course, and striving to achieve equitable health for all in a social context. Through this treatise we aimed at enriching and contributing to a global academic deliberation to encourage public health professionals to build a solid theoretical framework in our field.

## Methods

In this study we applied a narrative review approach to conceptually synthesize international scientific literature in examining the ethical foundations of the contemporary field of public health. In addition, we examined the existing international literature on public health values and principles. During this evolving process, we developed a framework of core ethical public health values and principles to be analyzed. More specifically, we used a narrative review to synthesize relevant literature, while conceptual analysis was done to examine relationships, similarities and differences among established ethical frameworks that could guide us for the selection and final integration of the most suitable and appropriate values and principles in the field of public health.

### Data source and search strategy

An extensive online search for publicly published articles and other sources of pertinent information was conducted in order to identify published reports within the article’s focus. A broad keyword strategy was developed for each group. Two independent search strategies focused on a comprehensive public health concept with specific terms for values and principles, respectively. Searches were applied to titles, abstracts and keywords and were limited to English-language journal articles and reviews with final publication status. The search was conducted separately for the two domains under investigation (values and principles). Within each dataset, duplicates were assessed using doi and title matching. Titles, abstracts, author keywords and index keywords were also screened manually. To expand the search, additional websites of health authorities, (such as the WHO, National Public Health Organizations, and other International Public Health Institutes and Associations) were screened. Finally, an extensive manual search for relevant scientific references from identified articles was also conducted.

Two researchers were assigned to review the international literature on values and principles, respectively. The first researcher focused on public health values, while the second researcher examined the literature on public health principles. Both researchers further examined the references of identified articles. Each researcher reviewed all identified articles. They then separately developed a set of values or principles that were deemed suitable for the treatise based on a narrative review synthesis. The guidance used for the selection process was based on the appropriateness of values and principles to support the development of public health programs for prevention and health promotion and enactment of relevant public health policies and legislation. The ultimate criteria and overarching umbrella paradigm used for the final selection were guided by the ability to advance the four goals of public health; namely whether the final selection of values and principles were strategically situated to (a) protect and promote health and wellbeing, (b) prevent disease and disability, (c) prolong life, and (d) maintain and improve quality of life.

The draft proposal developed by each researcher was reviewed by the junior and senior scientist of the team, who made adjustments on both values and principles based on their own review of the relevant scientific articles. The final framework of values and principles included in the current treatise was agreed by consensus between all co-authors and was significantly informed by established public health ethics, bioethics and human-rights literature.

## Core values

### Respect

The value of respect stands for the treatment of patients with dignity as people enjoy human rights. This means that public health professionals should respect autonomy of people, including patients and protect them from health-related challenges or even restore their autonomy in case of such challenge. Respect also involves recognition of the essential people’s rights to know, to privacy, to treatment. Individuals also deserve the right to make informed decisions regarding their health, while people with diminished autonomy (e.g., children, the older adults or people with disabilities) deserve additional support and protection as well as equal access to relevant services (19). Thus, informed consent stems from the main value of respect. Respect focuses on the right to self-determination and is necessary not only in individual healthcare delivery but also in the public health domain. Therefore, personal freedom should be respected, while providing the necessary protection for people who are incapable of making informed decisions (13).

By integrating respect into public health ethics, the intrinsic value of each person is enhanced, while the negligence of personal well-being in favor of wider community goals is being avoided. Crucially, whenever limitations on autonomy are proposed for the purpose of a greater public health benefit, the responsibility for providing overwhelming justification must lie upon those who endorse such actions. Adopting this approach guarantees a balanced and morally appropriate public health framework that respects individual freedom along with community benefits, while strengthening the informed decision-making process in dealing with vulnerable groups (14).

### Beneficence

The value of beneficence is defined as the act of contributing to the well-being of others as an essential moral value. In particular, health promotion becomes important in actively considering ways to increase beneficence. Comprehensive policies including environmental regulations and health promotion programs are facilitators in maximizing social benefits (20). It is the duty of health professionals to perform for the people’s good, which underlies a variety of ethical standards to protect and defend their rights, to prevent doing harm, aid those disabled, save people in danger and eliminate any conditions that are likely to lead to harm. The overall goal is to benefit people and promote their well-being (21). It demands that potential benefits for individuals and the community are maximized whilst potential harm is kept as low as possible. Beneficence implies a certain moral commitment to help others and promoting the common good (13).

In addition, the specific value concerns the role of advocate in defending human dignity on behalf of people unable to stand up for their rights and it is linked to the obligation of public health professionals and policy makers to share their skills and expertise to empower people in the field of health and public health. The large-scale strategies are targeted towards social determinants of health such as nutrition, housing and education to ensure sustainable and equitable services for all (19).

### Non-maleficence

The value of non-maleficence describes the responsibility of public health professionals to avoid harming people. Such value is consistent with general moral norms (do not kill/cause pain or suffering, do not cause injury or deprive others of life’s blessings) to the extent that we should avoid harming someone even if they ask for. Non-maleficence is the public health analogue of the Hippocratic oath stating that physicians should always strive to “first do no harm.” It is necessary for health professionals to assess the risks and benefits against various interventions. To be conscious of actions and/or inactions that influence health on a social frame. Large-scale interventions such as epidemiological surveillance, population screening, health regulations and population-based research need to balance public safety and individual rights (21). This value is useful in broad public health policies and practices, among which the degree of harm is “exchanged” for the evidence-based estimated possibility of causing greater harm or generating potential positive benefits (e.g., banning smoking in public places).

Challenges include the management of responses to pandemics, equitable distribution of resources and accountable health communication to avoid unnecessary fear (14). Nevertheless, according to Coughlin (13), non-maleficence does not eliminate the trade-off between potential harm and benefits since any intervention should be weighed against potential benefits to individuals and the broader public. Main concerns involve access to healthcare, environmental health impact, distribution of preventive programs and limited resources, and ensuring that vulnerable groups are not neglected in a discriminatory way.

### Justice

The value of justice is more generally related to balancing, to the maximum extent, the allocation of healthcare resources, treatment opportunities, social responsibilities as well as risks and benefits to guarantee equitable distribution among individuals and the society. Key features of this value include the efforts to avoid depriving someone of the benefits without strong justification and avoiding excessive burden imposed on others. The risks of denying healthcare to vulnerable groups are highlighted in this context. Also, justice is about avoiding discrimination with respect to age, gender, religion, social status, political affiliation, disability or mental disorder as well as equal access to resources, including preventive and therapeutic healthcare services (19). It is necessary for public health professionals to engage in activities that not only reduce existing health disparities but also those associated with “voice, power and wealth” as it concerns justice and equity in healthcare. This is needed due to disparities regarding social determinants since they can severely damage access to healthcare and negatively impact the health of individuals and populations. Environmental disparities demonstrate how low-income, and minority populations are experiencing health risks disproportionately due to systemic neglect (22).

### Solidarity

Solidarity is translated into mutual support among individuals, involving a commitment to promoting each other’s interests or group interests, even at a personal cost. In a setting based on solidarity, individuals not only share with others, in fact they are also allowed to receive something in return as an acknowledgement. Solidarity reflects the mutual dependence of people within a society, with public health policies such as universal healthcare and economic contributions ensuring equal access to such services being highlighted examples. Such measures protect everyone, since the benefits of public health are non-competitive and non-rivalrous given that health is considered a non-excludable access good (23). Also, solidarity emerges through the nature of human beings as both biological and social entities. Establishing and maintaining the value of solidarity has clearly a key role in contributing to the elimination of inequalities related to healthcare services and equal access to social determinants of health and well-being.

Solidarity is developed via the joint cognitive and emotional recognition of human dependencies. Solidarity is a complex term that attempts to incorporate the foundation of society practices from which moral values emerge. These values are produced and reproduced throughout time and mainly refer to the extent a society is united, connected and cooperative, sharing collective overt and covert benefits (24). A socially cohesive community means a place where cultural diversity is both tolerated and embraced, a society where the overwhelming number of people respect the law and human rights and is willing to share a common bond between social classes and other social rankings along with communal responsibility. Solidarity is also enhanced by social networks, shared resources and mutual support, where collaborative efforts, like local health initiatives and community-driven behaviors, benefit everyone creating an inclusive environment where healthcare resources are reachable by all (13).

Moreover, solidarity reflects public health’s engagement in community and population health. A community is made up of a web of social bonds which are held in place by family relations, neighborhood collaboration, common interests and strong connections of loyalty, friendship and empathy. Solidarity requires acknowledging the fact that we are all together when confronted by our greatest challenges and must be united to face them in a collaborative way (22).

## Core principles

### Accessibility

Accessibility is fundamental for public health, ensuring that individuals and communities have access to essential healthcare services and programs without unnecessary barriers. It determines whether people receive preventive care, timely medical attention, and participate in health interventions that can significantly improve health outcomes and reduce social disparities. Accessibility translates into reasonable access, which refers to timely and uniform access to healthcare services. When healthcare services are accessible, health equity improves and overall public health systems become more efficient and responsive to community needs. A key aspect of accessibility is preventive medicine which proactively reduces health disparities by making essential healthcare services widely available (25).

Accessibility is the backbone of effective public health infrastructures (26). Without it, healthcare becomes a privilege rather than a right, leading to exacerbation of health disparities, while undermining disease prevention efforts within society. Policies that remove barriers (financial, geographical or systemic), are essential in building an equitable health system, which serves all individuals, regardless of their background or economic status. By prioritizing accessibility, public health can truly fulfill its mission of promoting health, preventing disease and improving quality of life for all. Accessibility is deeply interconnected with affordability, as financial limitations remain the most significant barriers of healthcare access, particularly for low-income populations (27).

### Affordability

Affordability is fundamental for healthcare access and public health programs, ensuring that individuals can obtain necessary medical services and enroll in health promoting programs without facing financial difficulties. When healthcare costs are excessive, people are more likely to delay or neglect preventive services or essential treatments, leading to higher long-term expenses and worsened health outcomes. Preventive care is significantly more economical than treating diseases (28). Affordable means lowering high cost of personal expenses in health, removing transportation and geographic barriers and minimizing hidden costs of public health programs.

It is well documented that chronic non-communicable diseases such as cardiovascular disease, cancer and diabetes impose substantial economic costs. Public health campaigns that promote preventive strategies, such as healthier diets and physical activity, have also been shown to reduce treatment costs and alleviate financial strain on both individuals and healthcare systems. Universal health coverage and increased investment in preventive care are essential strategies for ensuring affordability. Reducing out-of-pocket expenses through government funding and public private partnerships can further alleviate the financial burden of individuals, making healthcare services more affordable (29).

Affordability is essential for an effective public health system. Without it, individuals face financial obstacles that prevent them from seeking necessary care, exacerbating health disparities and increasing overall healthcare costs. Policies that promote affordability through preventive care investments, cost sharing reductions and universal coverage schemes, are essential in creating a healthcare system that is both financially sustainable and accessible to all to improve health outcomes on a broader scale.

### Acceptability

Acceptability in public health refers to the extent which healthcare services align with the cultural, social, and ethical values of individuals and communities. A key aspect of acceptability is ensuring that healthcare services and public health programs are designed according to people’s values, preferences and needs. Effective public health interventions should take into consideration local beliefs, social norms and societal practices to ensure community participation. For instance, prenatal care and breastfeeding programs should respect cultural traditions, while community specific health education campaigns must adjust messages to the linguistic and cultural preferences of target populations. Vaccine hesitancy is another field where public perception is shaped by social and cultural beliefs, along with evidence-based outcomes necessitating culturally sensitive health communication campaigns. To enhance acceptability, public health policies should be respectful and adapted to local cultures through community engagement, culturally competent healthcare providers and inclusive policymaking. Addressing language barriers and respecting social and religious beliefs along with improving health literacy contribute to making healthcare an acceptable public good, which enhances service delivery, fosters greater public trust and participation and leads to improved health outcomes (30).

### Effectiveness

Effectiveness refers to the ability of public health interventions to produce measurable improvements in population health. Evidence-informed, cost-effective and robust policies and programs are fundamental in designing, implementing, assessing and improving public health interventions. Epidemiological studies track disease patterns and inform targeted interventions, which enhance their effectiveness. Health behavior interventions such as smoking cessation programs rely on behavioral change models that have been scientifically proven to reduce health risks. Additionally, public health metrics and biostatistics play a crucial role in evaluating intervention outcomes to ensure that policies are data driven. To maximize effectiveness, public health initiatives should be based on scientific research and continuously be evaluated and adapted according to real-time health trends (31).

Effectiveness is a fundamental principle in public health, ensuring that interventions produce measurable and lasting improvements in population health and should not be confused or equated with efficiency. By integrating evidence-based practices, data-driven decision making, and comprehensive healthcare strategies, public health approaches could improve overall community health outcomes (32).

### Participation

A participatory approach in public health is essential in facilitating active engagement of individuals and communities in the design of specific interventions in health through an inclusive decision-making process. Empowering local populations strengthens public health efforts, fosters sustainable change and increases participation. Community-based interventions, and economic and environmental factors also shape health outcomes, highlighting the importance of participatory policy making. An approach that involves community leaders and public health agencies ensures that health interventions are sustainable, widely accepted and aligned with community needs. By engaging individuals to share concerns and contribute to the design and development process, public health professionals empower communities to secure active participation (33). Incorporating participation into public health interventions enables policies and programs to generate community actions that are not only effective but also share long-term success.

## Discussion

By presenting an integrated framework of core public health values and principles, we are contributing to a recognized gap in international literature through the discussion of a theoretical foundation in a comprehensive manner. We finalized our work by delineating a framework that included repeated ethical domains as reported in the field of public health, which were applicable to both individual and population health. In addition, our work addresses vulnerable populations, universal coverage and equity with particular application in the development and implementation of public health policies and programs. The proposed framework builds on and does not simply reproduce prior ethical approaches.

Our five core values were captured partially in the four principles of Beauchamp and Childress’s principles of biomedical ethics (autonomy, beneficence, non-maleficence and justice) (34). The inclusion of solidarity extends this foundation by recognizing the mutual support and collective responsibilities for health that characterize population-level public health.

The five principles were subsequently examined in relation to the four dimensions of the AAAQ framework: availability, accessibility, acceptability and quality. In that instrument, “acceptability” means being respectful of medical ethics and culturally appropriate, while “accessibility” explicitly encompasses non-discrimination and economic accessibility (35). In our proposed framework we treat affordability as an explicit and independent principle, rather than subsuming economic accessibility within the broader concept of accessibility. Moreover, Childress et al. (36) set out general moral considerations including five justificatory conditions, one of which is effectiveness, itself one of our treatise’s five principles. Finally, participation emphasizes the active community involvement in public health decision-making. Thus, through the proposed framework we do not claim to replace AAAQ but seek to complement it by connecting service- and rights-based requirements with broader ethical values and practical applications in public health policy.

Through our conceptual synthesis, we have positioned the proposed ten-construct framework within the existing ethical and human-rights approaches, rather than presenting it as a new development. Its distinctive and novel contribution lies in the integration of the five values and five principles, thereby linking the moral justification of public health with relevant actions based on solid criteria. The framework further places particular emphasis on collective responsibility, equity, social vulnerability and community participation, dimensions that are not captured in the same integrated way by previous reports.

An illustrated example of the application of the proposed framework could be the problem of childhood obesity where respect for children, subjects with limited autonomy, is of paramount importance. Beneficence and non-Maleficence becomes a very sensitive issue for public health interventions among children along with justice and solidarity. Investing time and effort along with financial resources in policies and programs to curb childhood obesity should definitely be based on accessibility, affordability and acceptability to avoid exclusion of groups of children. Finally, effective, data-driven and participatory approaches are required to achieve meaningful positive outcomes in such a difficult and complex public health problem. Due to the extent of the current treatise we are restricted to further expand on the details of applications of our framework in the childhood obesity example.

A highlighted example of these principles in action is the Patient Protection and Affordable Care Act (ACA), which illustrates how principles can be embedded into public health legislation and policies to increase system efficiency and improve health outcomes (37). For instance, the ACA promotes non-discriminatory healthcare policies, expands preventive health services and introduces initiatives aimed at making healthcare more patient oriented. A key feature of ACA, the National Prevention Strategy, focuses on reducing the risks of chronic diseases through community-based interventions. This strategy acknowledges that healthcare must be socially inclusive and culturally sensitive to be truly effective. Additionally, the ACA sets nutrition labeling requirements and wellness program motives to encourage health-conscious behaviors, making healthcare more accessible, affordable and acceptable to diverse populations (38).

According to Chait and Glied (39), ACA significantly advances public health by improving affordability, acceptability, effectiveness, and participation. It reduces financial and geographic barriers to care through Medicaid expansion, making preventive services more accessible and lowering long-term healthcare costs. The ACA also emphasizes culturally tailored interventions and addresses social determinants of health, such as food insecurity and nutrition in schools, enhancing the acceptability of public health programs.

In addition, evidence-based initiatives like the National Diabetes Prevention Program and the Tips from Former Smokers campaign demonstrate measurable success in improving health outcomes through a concerted community effort. Crucially, the ACA promotes community participation by funding partnerships in 34 states in the United States and supporting workplace wellness programs, ensuring that public health policies are community-driven, sustainable, and aligned with local needs.

A systematic review conducted by Haldane el.al. (40) provides compelling evidence linking the proposed principles to improved outcomes in community participation in health service development, implementation and evaluation. Synthesizing findings from 49 studies across high and upper middle-income countries, the review demonstrates that these core values are instrumental in driving success in both the process and outcome measures of public health programs. Integrating the proposed principles is critical for shaping an equitable and effective public health system. In the face of rising healthcare costs, widening health disparities and workforce shortages, appropriate policy formulation and implementation becomes a necessary approach (27).

Furthermore, a scoping review on the application of primary healthcare principles in National Community Health Worker (CHW) programs in low- and middle-income countries demonstrates how these principles are integrated into public health initiatives and have a measurable impact on health outcomes. The study explores the extent to which elements such as universal health coverage, community participation and intersectional coordination are reflected in CHW programs, and how these factors influence healthcare access and quality in resource-constrained settings (41).

The implementation of these principles is also evident in White’s article (2015), which offers a strong foundation for understanding how primary healthcare and public health support these principles, by advocating for integrated universal health coverage systems that prioritize prevention, equity and community engagement (42, 43).

Ultimately, the values and principles of public health are essential in creating equitable and effective healthcare systems. The study on Specific Timely Appointments for Triage (STAT) offers another tangible example of how these principles can be applied to improve healthcare delivery and health outcomes. By emphasizing on accessibility, affordability, acceptability, effectiveness and participation, public health could build systems that ensure better health for all (44).

In relation to the principle of participation, case studies such as the Communities That Care initiative (45) and public health interventions in chronic disease management (46, 47) demonstrate that true participation is not merely a recommendation but active engagement in decision making and implementation. Community driven health initiatives ensure long-term success by fostering community empowerment, building trust and creating resilient health systems (48).

The establishment and operation of national public health agencies and organizations as well as of healthcare facilities constitute the most efficient way to implement public policy and population-level programs in the field of public health. Epidemiology strengthens healthcare accessibility by identifying disease patterns, tracking outbreaks and enabling timely interventions. Epidemiological surveillance, by continuous monitoring of public health threats ensures that healthcare systems respond swiftly especially in marginalized communities, where access to healthcare may already be limited. However, improving accessibility requires more than just basic infrastructure including targeted policies that remove financial, geographical and systemic barriers. Mobile health service programs and telemedicine appear to be additional tools in extending public health programs and healthcare services to those who would otherwise face significant barriers (49).

As mentioned before, accessibility is deeply interconnected with affordability and financial limitations remain the most significant barriers of healthcare access, particularly for low-income populations. Public health policies should address economic inequalities and implement financing models that do not exclude those most in need. Healthcare services are also expected to be culturally and ethically suitable for the communities they serve. Policies should be designed with sensitivity to diverse values and beliefs, gaining trust and encouraging greater utilization of healthcare services.

Universal health coverage is a critical solution for achieving equitable healthcare access, as outlined by the WHO. Reduction of out-of-pocket expenses and implementation of mandatory progressive prepayment systems are essential strategies for structuring healthcare financing in a way that prioritizes prevention and reduces economic disparities. By adopting these approaches, healthcare systems could ensure that essential services remain financially accessible to all individuals, regardless of socioeconomic status (42).

The inclusion of cultural competence, community involvement, and community-centered approaches, leads healthcare systems to strengthen service delivery, build greater public trust and participation and improve health outcomes – as they reflect on all public health values and principles incorporated into real-world practice. Data driven policy making is another critical aspect, as public health metrics and continuous evaluation ensure that interventions remain adaptable and effective. Healthcare policies must be guided by scientific research and public health data, to achieve sustainable health improvements. Effective public health programs require policies that encourage community participation, empower local health workers and foster cross-sectoral collaborations to address broader determinants of health, leading to lasting improvements in health outcomes (50).

## Limitations

We would like to acknowledge several limitations. First, as a narrative review, the identification and interpretation of the literature involved a degree of author judgement and may therefore be subject to selection and interpretive bias. Second, the field already has a developed body of public-health-specific frameworks (34–36, 51). Several proposed values and principles overlap with already established frameworks, including those in the above citations and the AAAQ/right-to-health framework. Consequently, our final framework should be viewed as a synthetic approach rather than as a claim that each component is entirely novel. Finally, the proposed framework may require contextual adaptation because the interpretation and relative weighting of ethical values and principles could vary across cultural, socioeconomic and health-system settings. Future research should therefore examine the framework across diverse public health settings and evaluate its practical utility in public health programs and policy decision-making. Due to the extensive discussion, we were not able to highlight the global importance of our proposed framework with additional international examples.

## Conclusion

Although a wide diversity of theories has been proposed in the field of public health, the proposed framework of the core public health values—respect, beneficence, non-maleficence, justice, and solidarity—and the core principles—accessibility, affordability, acceptability, effectiveness, and participation—discussed in the current treatise, is aligned with the foundation of a successful public health paradigm as depicted in Figure 1. These core values guide the development and implementation of policies and programs by ensuring dignity and fairness, promoting well-being, preventing harm, and fostering a collective commitment to health equity. The core principles shape public health policies and preventive medicine, promoting equitable access, cost effectiveness, evidence-based programs, cultural relevance and community involvement to improve population health and reduce disease burden. By embedding these values and principles into public health policies and practice, public health professionals can uphold ethical standards that protect individuals and communities, support social justice, and encourage shared responsibility for improving population health outcomes. Ultimately, these values and principles create a comprehensive framework that is both ethically grounded, socially adapted and practically oriented towards achieving equitable, responsive and sustainable healthcare systems that strengthen community capacity and trust leading to sustainable public health outcomes.
